# Global Mapping of Interventions to Improve Quality of Life of People with Diabetes in 1990–2018

**DOI:** 10.3390/ijerph17051597

**Published:** 2020-03-02

**Authors:** Bach Xuan Tran, Long Hoang Nguyen, Ngoc Minh Pham, Huyen Thanh Thi Vu, Hung Trong Nguyen, Duong Huong Phan, Giang Hai Ha, Hai Quang Pham, Thao Phuong Nguyen, Carl A. Latkin, Cyrus S.H. Ho, Roger C.M. Ho

**Affiliations:** 1 Institute for Preventive Medicine and Public Health, Hanoi Medical University, Hanoi 100000, Vietnam; 2 Bloomberg School of Public Health, Johns Hopkins University, Baltimore, MD 21205, USA; carl.latkin@jhu.edu; 3 Department of Public Health Sciences, Karolinska Institutet, 17177 Stockholm, Sweden; hoang.nguyen@ki.se; 4 School of Public Health, Faculty of Health Sciences, Curtin University, Perth, WA 2605, Australia; minh.pn@tnu.edu.vn; 5 Thai Nguyen University of Medicine and Pharmacy, Thai Nguyen 250000, Vietnam; 6 Department of Gerontology and Geriatrics, Hanoi Medical University, Hanoi 100000, Vietnam; vuthanhhuyen11@hmu.edu.vn; 7 Scientific Research Department, National Geriatric Hospital, Hanoi 100000, Vietnam; 8 Clinical Nutrition and Dietetics Department, National Institute of Nutrition, Hanoi 100000, Vietnam; nguyentronghung9602@yahoo.com; 9 National Hospital of Endocrinology, Hanoi 100000, Vietnam; phanhuongduong@gmail.com; 10 Institute for Global Health Innovations, Duy Tan University, Da Nang 550000, Vietnam; hahaigiang@duytan.edu.vn (G.H.H.); phamquanghai@duytan.edu.vn (H.Q.P.); 11 Faculty of Pharmacy, Duy Tan University, Danang 550000, Vietnam; 12 Faculty of Medicine, Duy Tan University, Danang 550000, Vietnam; 13 Center of Excellence in Evidence-based Medicine, Nguyen Tat Thanh University, Ho Chi Minh City 700000, Vietnam; thao.coentt@gmail.com; 14 Department of Psychological Medicine, National University Hospital, Singapore 119074, Singapore; cyrushosh@gmail.com; 15 Center of Excellence in Behavioral Medicine, Nguyen Tat Thanh University, Ho Chi Minh City 700000, Vietnam; pcmrhcm@nus.edu.sg; 16 Department of Psychological Medicine, Yong Loo Lin School of Medicine, National University of Singapore, Singapore 119228, Singapore; 17 Institute for Health Innovation and Technology (iHealthtech), National University of Singapore, Singapore 119077, Singapore

**Keywords:** scientometrics, content analysis, text mining, interventions, diabetes, QOL

## Abstract

Improving the quality of life (QOL) of people living with diabetes is the ultimate goal of diabetes care. This study provides a quantitative overview of global research on interventions aiming to improve QOL among people with diabetes. A total of 700 English peer-reviewed papers published during 1990–2018 were collected and extracted from the Web of Science databases. Latent Dirichlet Allocation (LDA) analysis was undertaken to categorize papers by topic or theme. Results showed an increase in interventions to improve the QOL of patients with diabetes across the time period, with major contributions from high-income countries. Community- and family-based interventions, including those focused on lifestyle and utilizing digital technologies, were common approaches. Interventions that addressed comorbidities in people with diabetes also increased. Our findings emphasize the necessity of translating the evidence from clinical interventions to community interventions. In addition, they underline the importance of developing collaborative research between developed and developing countries.

## 1. Introduction

Diabetes mellitus is well recognized as a global public health crisis. It is a chronic metabolic disorder characterized by elevated blood glucose levels due to the body’s impaired insulin secretion and/or insulin resistance [1]. Persistent diabetes devastates vascular and nerve systems, causing many severe life-threatening complications (e.g., cardiovascular diseases, neuropathy, diabetic foot complications, diabetic retinopathies, or renal failure) and increasing the risk of hospitalization and mortality [2,3,4]. This disease has now been among the leading causes of disease burden worldwide [5]. According to global estimates, in 2017, over 451 million people were reported to be living with diabetes, with more than 5 million diabetes-related deaths [1]. 

Like other chronic diseases, diabetes cannot be completely cured [6]. Therefore, ensuring that people living with diabetes have a good quality of life (QOL) and can function adequately has become the ultimate goal of diabetic care [7]. Research increasingly looks to QOL as a favorable outcome of interventions focused on diabetes [2,6]. QOL is a multidimensional concept that does not have a unified definition [8,9,10,11]. The World Health Organization defines QOL as “an individual’s perceptions of their position in life, in the context of the culture and value systems in which they live, and in relation to their goals, expectations, standards, and concerns” [11]. When two treatments have similar clinical outcomes, measuring QOL can reflect patients’ different experiences or perceptions of treatments and symptoms, helping clinicians to identify which intervention’s benefits outweigh its drawbacks [12]. In the case of diabetes, evaluating QOL is critically important as its enhancement is associated with good self-care management, including adherence to prescribed medication and suggested lifestyle modifications, which are significant protective factors for diabetes care [2,6,13,14]. 

A growing body of literature (including trials, systematic reviews, and meta-analyses) examines the effectiveness of various approaches (from health education to behavioral modification, pharmacotherapy, and surgery [12,15,16,17,18]) in enhancing treatment outcomes and QOL among people with diabetes. However, few publications feature updated quantitative data (i.e, bibliometric or scientometric analysis) focusing on interventions aimed at improving QOL in people living with diabetes. Recently, most of the published bibliometric studies have concentrated on diabetes in general [19,20,21], diabetic complications [22] or comorbidities [23], and the use of specific therapy in diabetes treatment [24]. Looking at these offers a comprehensive picture of the current approaches utilized for improving QOL, the status of international collaboration, and the gap between high- and low-income nations, which is vital in developing a roadmap for a global research agenda that will help optimize diabetes treatment outcomes. The aim of this study is thus to assess the outcomes of recent interventions to improve QOL of people with diabetes.

## 2. Materials and Methods 

### 2.1. Searching Strategy

We performed a combined bibliometric and content analysis of publications covering the interventions to improve QOL among people with diabetes. The Web of Science (WOS) Core Collection was selected for the retrieval of data from 1900 to 31 December, 2018. The reasons for selecting the WOS include the availability of necessary information for analyzing contents of papers such as names and addresses of authors, titles/abstracts of articles, keywords, total citations and downloads, and research area coverage, which is far more than other accessible databases. Moreover, this database has a high citation report coverage and supports various analysis measures that facilitate bibliometric analysis of the existing literature [25,26]. 

Articles were included if they (1) involved interventions (randomized controlled trials [RCTs], pre-post or quasi-experiments); (2) focused on people with diabetes as the targeted population; (3) had QOL or health-related QOL as primary or secondary outcomes; (4) were original articles; (5) were published in English scientific journals indexed in the WOS; and (6) had comprehensive information on the authors. 

We excluded gray literature (e.g., reports, dissertations, theses, letters, news, etc.), book and book chapters, and conference abstracts/proceedings because some of these might have been published as scientific papers in peer-reviewed journals which could cause duplications. Papers without author information were also excluded because they could not be used to analyze the affiliation and collaboration networks across countries. Additionally, papers about narrative reviews/systematic reviews/meta-analysis studies were excluded because they were not original studies, which might not reflect the tendency of research development. Study protocols of interventions or papers reporting only baseline characteristics were not eligible because they did not assess the effect of specific interventions on the QOL.

To identify relevant articles, we performed the search strategy as follows:

- First, we produced a QOL dataset by employing topic search terms such as “quality of life” and “well-being” on the WOS. Among 441,617 records after searching, we excluded 114,212 documents (including: 4364 papers that were published in 2019; 25,543 documents that were non-English articles; 84,083 documents that were not articles/reviews; and 222 documents that had insufficient author information). A total of 327,405 quality of life-related papers were used for the next step.
- Second, we filtered the papers regarding interventions in diabetes populations by using a set of title/abstract terms related to “diabetes” (AND “intervention” OR “trial”) and saved it as the final dataset. A total of 323,079 papers were excluded, resulting in 4326 papers that were included in the next phase.
- Finally, we screened 4326 papers by reading their titles and abstracts and excluded 3626 papers that were not eligible according to the inclusion and exclusion criteria.

Full records of articles were exported and downloaded independently by two members in the research team. The third researcher performed a cross-check between two datasets to ensure their consistency. 

### 2.2. Data Analysis

STATA version 15.0 (STATA Corp., TX, USA) was utilized for data analysis. We performed a descriptive analysis using the following indicators: publication year, the number of papers per country/per year, total citations up to 2018, mean citation rate per year, total usage in the last six months/five years, and mean use rate in the last six months/five years. The VOSviewer (version 1.6.8, Center for Science and Technology, Leiden University, the Netherlands) was employed to illustrate the co-occurrence of the most frequent terms in titles and abstracts. Country collaboration networks were illustrated by using the Circos platform [27]. As for content analysis, we analyzed the hierarchical clustering of major research disciplines in the interventions and visualized it in a Dendrogram. Thematic analysis was performed using the Latent Dirichlet Allocation (LDA) technique, which supports classified papers in ten major themes/topics [18,28,29,30,31]. Titles and abstracts of papers in every topic/theme were then reviewed by two researchers. Any disagreements were addressed by discussing it with a senior researcher. We then calculated the number of papers per topic and determined any change in research interests by ranking the total number of publications per topic in the past five years (from 2013–2018). 

## 3. Results

**Figure 1:**
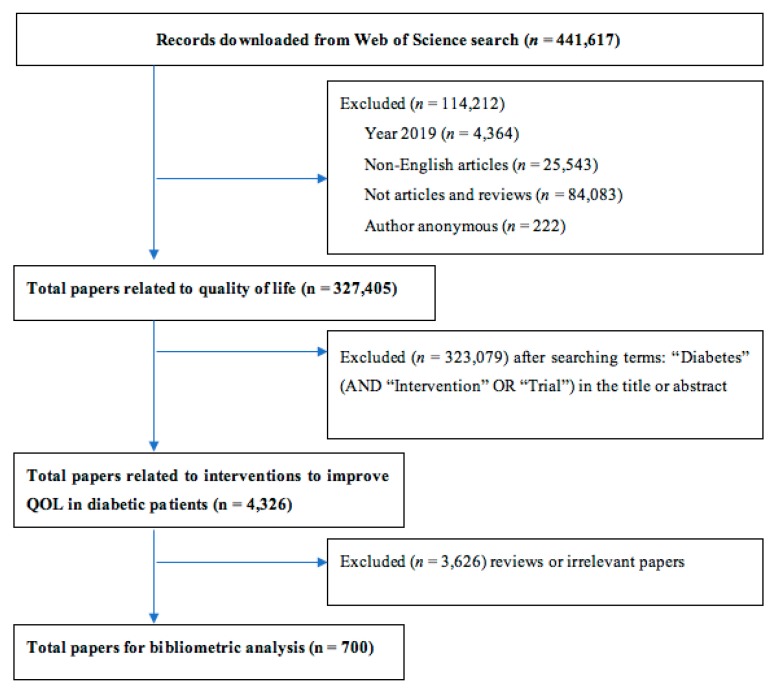
Selection of papers.

Figure 1 illustrates the searching process. Among the 327,405 papers on QOL, 700 papers on interventions to improve QOL of people with diabetes were selected as eligible.

Table 1 shows that in the period 1990–2018, there were a total of 700 papers published about interventions to improve the QOL among people with diabetes. From the first paper counted in 1990, the volume of annual articles increased significantly over time to reach a peak of 67 papers in 2015, before falling to 63 papers by the end of 2018. Papers published in 2005 and 1998 had the highest mean citation rate per year (9.7 and 9.0, respectively). Articles published in 2015 and 2005 had the highest total usage (i.e., the total number of downloads) and mean use rate in the last five years, respectively. In the last six months, the total usage and the mean use rate of papers published in 2018 were higher than those published in other years. 

Overall, 700 papers were published by authors in 61 countries. The United States (U.S.) and the United Kingdom (U.K.) contributed the most publications (322 and 160 papers, respectively), followed by Germany (101 papers), Australia (87 papers), and the Netherlands (85 papers). Only China was the only middle-income country in the top ten nations with the greatest number of publications, while the others were high-income nations. Meanwhile, among the top 20 countries, only China, India, Malaysia, and Iran were middle-income countries. 

**Figure 2:**
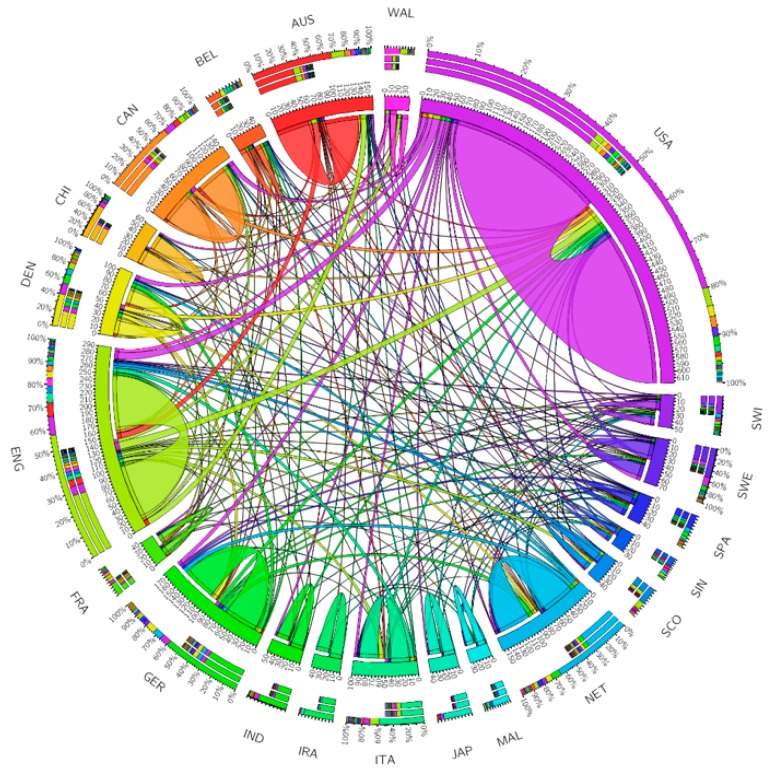
Collaboration network between the top 20 countries by the number of publications. The outer rim reflects the volume of collaborations between each country and the other countries in the top 20, showing collaboration among countries. Abbreviation: USA, the United States of America; ENG, England; GER, Germany; AUS, Australia; NET, the Netherlands; CAN, Canada; DEN, Denmark; ITA, Italy; SWE, Sweden; CHI, China; IND, India; SWI, Switzerland, BEL, Belgium; SPA, Spain; MAL, Malaysia; FRA, France; JAP, Japan; SCO, Scotland; IRA, Iran; SIN, Singapore.

Figure 2 depicts networks of collaboration among the top 20 countries having the highest volume of publications. The U.K. and the U.S. had the highest amount of collaborations, with 28 and 25 countries, correspondingly. In the U.S., among 322 published papers, there were more than 610 affiliations mentioned. Approximately 80% of them were from U.S. authors (~490 affiliations), 4% were from the U.K. (~23 affiliations), and 2% were from Denmark (~10 affiliations). Similarly, in the U.K., 160 papers were products of authors from 290 affiliations (or organizations), with ~58% from the U.K., 8% from the U.S. and 6% from Australia. These countries were followed by Germany (22 countries), Denmark (18 countries), and the Netherlands (18 countries). 

**Figure 3:**
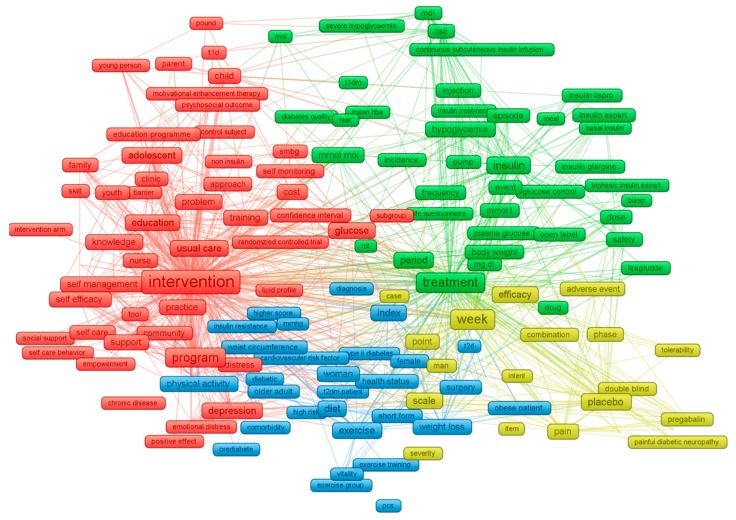
Co-occurrence of most frequent terms in titles and abstracts. The colors of the nodes indicate principal components of the data structure; the node size was scaled to the keywords’ occurrences; the thickness of the lines was drawn based on the strength of the association between two keywords.

To illustrate the scope of the selected studies, we performed a content analysis to evaluate the co-occurrence of the most frequent terms in the abstracts and titles. Figure 3 shows four major clusters emerging from the 271 most common keywords with a co-occurrence rate of at least 15: (1) the yellow cluster refers to clinical trials to test the efficacy and safety of drugs, as well as to control pain and overweight/obesity among people with diabetes (e.g., placebo, efficacy, safety, dose, pain, body weight, obesity, weight loss); (2) the blue cluster indicates trials focusing on diabetes prevention such as lifestyle- or behavior-related interventions (e.g., physical activity, exercise training, diet, etc.); (3) the green cluster covers the interventions using insulin-related therapies to control blood glucose level, particularly among patients with type 1 diabetes (e.g., insulin treatment, insulin glargine, etc.); and (4) the red cluster refers to interventions in the community to promote self-care capability (e.g., self-care, practice, self-efficacy, caregivers, etc.), as well as reduce the risk of psychological problems (e.g., depression or distress) among people with diabetes, especially in adolescents and children (e.g., adolescent, child, parent).

The top 10 clinical trials having the highest number of citations are presented in Table 2. All of them were RCTs, which focused mainly on the effectiveness of different medications, surgery, or behavioral therapies in controlling blood glucose; reducing the negative effects of diabetic complications (such as diabetic neuropathy, obesity, limb ischemia, or perinatal complications); and improving QOL in people with diabetes, especially pregnant women, children, and adolescents. Among the top three papers, the first paper, by Crowther et al., titled “Effect of treatment of gestational diabetes mellitus on pregnancy outcomes,” published in 2005 in the *New England Journal of Medicine,* had the highest number of citations and evaluated the efficacy of dietary advice, blood glucose monitoring, and insulin therapy (by comparing the intervention group to a control group receiving only routine care) in treating gestational diabetes in pregnant women to prevent perinatal complications [32]. By using the Short-form 36 (SF-36) instrument, the results of this study showed a significant increase in QOL among participants [32]. The second paper, titled “Gabapentin for the symptomatic treatment of painful neuropathy in patients with diabetes mellitus—A randomized controlled trial,” also used SF-36 and showed that gabapentin use significantly improved the QOL of patients with diabetic neuropathy compared to the placebo group [33]. The SF-36 was also used in the third-most cited paper, entitled “Bariatric Surgery versus Intensive Medical Therapy for Diabetes-3-Year Outcomes” [34]. The authors of this study indicated that bariatric surgery significantly enhanced health-related QOL among obese patients with type 2 diabetes compared to those receiving intensive medical therapy only [34].

**Figure 4:**
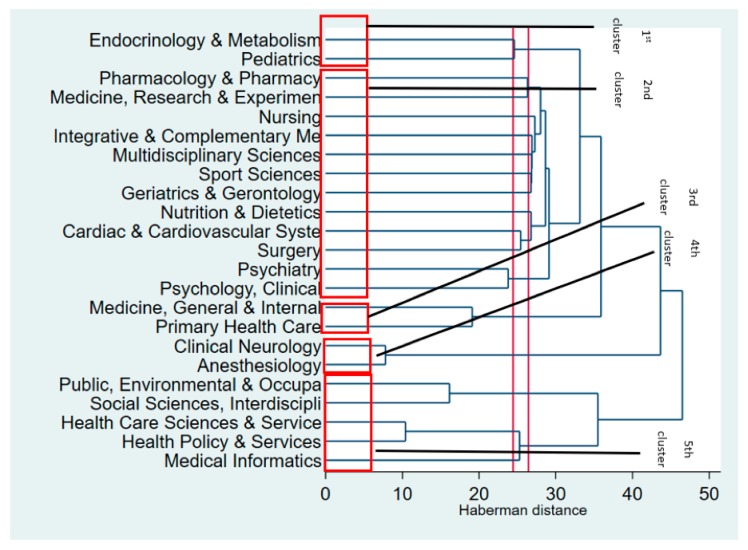
Dendrogram of coincidence of research areas using WOS classifications.

Figure 4 presents the hierarchical clustering of major research disciplines in interventions aiming to improve the QOL of people with type 2 diabetes. The horizontal axis reflects the dissimilarity between clusters, while the vertical axis reveals disciplines of pooled papers. This figure shows that interventions were separated into five major clusters. In the first cluster, the “Pediatrics” grouping is connected to “Endocrinology and Metabolism,” suggesting that the majority of interventions for children with diabetes and adolescents were concentrated on using medications to enhance metabolic pathways such as insulin infusion or an insulin pump. 

Meanwhile, the second cluster indicated clinical and lifestyle interventions to improve QOL in people with diabetes in general or with multiple comorbidities (such as cardiac disease and mental problems). For example, the “Pharmacology and Pharmacy” grouping was joined with “Medicine, Research and Experiment,” which indicated that interventions using a pharmacological approach such as fenofibrate, ranolazine, or other drugs in glycemic control, reduced the damage done by diabetes and its complications and improved QOL. In addition, the “Sport Sciences” discipline was combined with the “Geriatrics and Gerontology” disciplines, showing that the common intervention approaches in older people with diabetes were facilitating physical activity. Similarly, for people with diabetes suffering cardiac illnesses (“Cardiac and Cardiovascular System”), “Surgery” and “Nutrition and Dietetics” were the two most common interventions. 

The third cluster reveals clinical interventions to improve the QOL of people with diabetes in primary care settings. These included home- or community-based interventions. 

Meanwhile, the fourth cluster indicated clinical interventions to enhance the QOL of people with diabetes suffering from neurological pain. 

Finally, the fifth cluster showed public health, health service, health policy, and medical-information-technology-related interventions aiming to improve the QOL of people with diabetes. Notably, this cluster is not close to other groupings in the figure.

By using LDA, we categorized the selected interventions under ten major topic headings. The three topics with the highest number of publications included: (1) community-, family-, and eHealth-based interventions to improve self-management and self-efficacy; (2) lifestyle (e.g., physical activity and dietary) interventions in people with diabetes; (3) interventions focused on comorbidities in people with diabetes (Table 3). The first topic was investigated in 187 papers, accounting for 26.7% of total papers. Meanwhile, the second and third topics accounted for 17.7% and 15.3%, respectively. 

**Figure 5:**
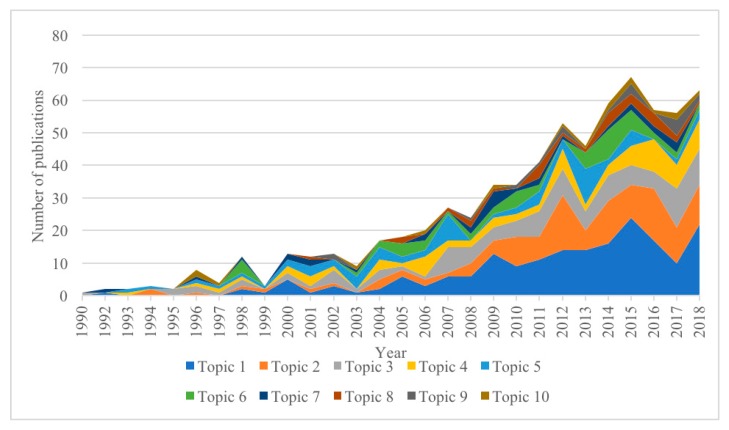
Changes in research topic development.

Figure 5 shows the correlation of publications to the ten topics. Recently, topics 1 and 2 received the greatest attention, with the largest number of publications every year in the three years 2015–2018. In the same period, the number of publications on topics 3 and 4 also increased, while the volume of publications on other topics decreased.

## 4. Discussion

Our study presents an overview of global research on interventions supporting QOL among people with diabetes during the period 1990–2018. The findings of this study indicated a general increase in the overall number of articles about this issue over time, and a significant disparity in the contributing countries based on income level. Community- and family-based interventions, including those focused on lifestyle and those utilizing digital technologies, were common approaches. Interventions that addressed comorbidities in people with diabetes also increased over the period. Our findings emphasize the necessity of translating the evidence from clinical interventions to community interventions. In addition, they underline the importance of developing collaborative research between high-income countries (HICs) and low- and middle-income countries (LMICs). It is particularly true since LMICs are predicted to experience a substantially greater diabetes burden in the coming years [41]. 

Diabetes is a chronic condition and requires good self-management and long-term metabolic control for patients’ QOL to improve [42]. Our analysis confirmed the findings of previous reviews [43] regarding approaches seen to increase the QOL of people with diabetes, including pharmacotherapy, surgery, and educational or lifestyle interventions to control blood glucose or diabetic complications, in both online (e.g., Internet or telephone) and offline settings (e.g., hospital, community, family, or school). Amid the increase in the diabetes burden as well as its pervasiveness in communities around the world, there is no doubt that community-, family-, or even online-based interventions are important to consider. This is confirmed by the high and growing number of publications on them, relative to other topics in the healthcare sector. We found that most studies focused on improving the self-management and self-efficacy of patients with diabetes, helping them monitor and control their blood glucose levels, as well as prevent the onset of diabetic complications [44]. This topic is followed by the application of lifestyle interventions (including diet with active physical activity), which plays a major role in enhancing diabetes outcomes and improving QOL. An earlier review of this type of intervention showed that it was more effective than pharmacotherapy in alleviating diabetic symptoms and complications, as well as in preventing the onset of diabetes [45]. The remaining topics included interventions to control and treat comorbidities and other complications such as neuropathic pain, mental disorders, cardiac diseases, and functional impairment such as foot ulcers or sexual disfunction. It has been confirmed in prior reviews that these problems significantly affect the QOL of people with diabetes [42,46]; therefore, medical approaches to solving these issues are critical to improving QOL not only in hospital settings but also in primary care and community settings. 

Notably, this study revealed a skewed geographical distribution of the selected publications toward HICs such as the United States, England, Germany, Australia, and the Netherlands. China was the only middle-income country among the top 10 active nations. However, it should be noted that the burden of diabetes has been much greater for LMICs than for HICs. In 2016, a global report showed that among the 425 million people living with diabetes, approximately 80% of them resided in LMICs, and this figure is expected to increase significantly in the coming decades [1]. This disparity was in line with a previous bibliometric study about diabetes, depression, and suicide, though these same countries had the highest volume of publications on this topic [23]. Other scientometric studies also underlined the insufficient contribution of LMICs to research, which does not help diminish the diabetes burden in these nations [19,20,47,48]. The concept of QOL refers to the individual’s perception of physical, psychological, and social conditions in a specific context and culture [11]. Thus, more contextualized evidence about the effects of interventions on the QOL of people with diabetes in LMICs is required to increase the applicability of these interventions. Insufficient evidence from LMICs might be due to a lack of funding sources, human resources, and infrastructure for research institutions [47,48,49]. This gap may not be fulfilled in the short term, but these countries might benefit from using evidence from neighboring nations having similar cultural, socioeconomic, and value systems. Regional collaborative networks may be developed such that LMICs with high research productivity (such as China and India) play a central role, with support from HICs. Such initiatives could strengthen the research capacity of member countries as well as increase the quality of evidence, which would serve as a foundation for further strategies to increase QOL of people with diabetes. 

This study has several limitations. First, our sample was limited to English-language publications indexed in the WOS database. This may not reflect the development of publications on interventions improving QOL among people with diabetes in countries where English is not a native language. Therefore, publications from these countries, many of them LMICs, might be underestimated. Additionally, we did not use the full text of publications for our content analysis. That said, we analyzed various levels of data, including keywords, the text of the title and abstract, and research discipline categories, and also employed advanced analytical methods such as LDA [50].

## 5. Conclusions

Global research on interventions to improve the QOL of people with diabetes gradually increased in recent decades, with major contributions from HICs. Community- and family-based interventions, including those focused on lifestyle and utilizing digital technologies, have been common. Interventions to address comorbidities in people with diabetes have also been on the rise. More contextualized interventions in LMICs, as well as regional initiatives to support LMICs in translating worldwide evidence, should be facilitated to alleviate the diabetes burden in these countries.

## Figures and Tables

**Table 1:** General characteristics of publications analyzed.

Year Published	Total Number of Papers	Total Citations	Mean Cite Rate Per Year	Total Usage Last 6 Months ^1^	Total Usage Last 5 Years ^2^	Mean Use Rate Last 6 Months	Mean Use Rate Last 5 Years
2018	63	158	2.5	218	405	3.5	1.3
2017	56	556	5.0	95	494	1.7	1.8
2016	57	455	2.7	51	601	0.9	2.1
2015	67	1009	3.8	77	1,135	1.1	3.4
2014	59	1845	6.3	67	927	1.1	3.1
2013	46	1205	4.4	25	808	0.5	3.5
2012	53	1342	3.6	28	871	0.5	3.3
2011	41	1127	3.4	16	501	0.4	2.4
2010	33	1020	3.4	24	380	0.7	2.2
2009	34	1590	4.7	23	370	0.7	2.2
2008	24	1372	5.2	10	297	0.4	2.5
2007	27	1244	3.8	18	308	0.7	2.3
2006	20	1038	4.0	8	156	0.4	1.6
2005	18	2438	9.7	18	335	1.0	3.7
2004	17	930	3.6	3	94	0.2	1.1
2003	9	870	6.0	3	79	0.3	1.8
2002	13	1233	5.6	1	79	0.1	1.2
2001	12	978	4.5	0	65	0.0	1.1
2000	13	1355	5.5	8	140	0.6	2.2
1999	3	202	3.4	1	9	0.3	0.6
1998	12	2265	9.0	1	98	0.1	1.6
1997	4	208	2.4	2	14	0.5	0.7
1996	8	1177	6.4	4	60	0.5	1.5
1995	2	202	4.2	2	13	1.0	1.3
1994	3	220	2.9	2	10	0.7	0.7
1993	2	13	0.3	0	0	0.0	0.0
1992	2	171	3.2	0	12	0.0	1.2
1990	1	37	1.3	0	0	0.0	0.0

^1^ Total usage: Total number of downloads. ^2^ Use rate: Total number of downloads/total number of papers.

**Table 2:** Top 10 most cited clinical trials.

Title	Journal	Total Citations	Publication Year	Cite Rate	Study Design	Type of Diabetes	Type of Interventions	QOL Tool
Effect of treatment of gestational diabetes mellitus on pregnancy outcomes [32]	New England Journal of Medicine	1,516	2005	108.3	RCT	gestational diabetes mellitus	dietary advice, blood glucose monitoring, and insulin therapy	SF-36
Gabapentin for the symptomatic treatment of painful neuropathy in patients with diabetes mellitus – A randomized controlled trial [33]	Journal of the American Medical Association	981	1998	46.7	RCT	Type 1 and type 2	Gabapentin	SF-36
Bariatric surgery versus intensive medical therapy for diabetes – 3-year outcomes [34]	New England Journal of Medicine	798	2014	159.6	RCT	Type 2	Bariatric Surgery, Intensive Medical Therapy	RAND-36
Does increased access to primary care reduce hospital readmissions?	New England Journal of Medicine	504	1996	21.9	RCT	General	Access to Primary Care	SF-36
Effectiveness of the diabetes education and self-management for ongoing and newly diagnosed (DESMOND) programme for people with newly diagnosed type 2 diabetes: Cluster randomized controlled trial [35]	British Medical Journal	356	2008	32.4	RCT	Type 2	Group education programme	WHOQOL-BREF
Double-blind randomized trial of tramadol for the treatment of the pain of diabetic neuropathy [36]	Neurology	351	1998	16.7	RCT	Type 1 and type 2	Tramadol	MOS
Controlled-release oxycodone relieves neuropathic pain: A randomized controlled trial in painful diabetic neuropathy [37]	Pain	324	2003	20.3	RCT	Type 1 and type 2	Controlled-release oxycodone	SF-36
Randomized placebo-controlled clinical trial of Lorcaserin for weight loss in Type 2 diabetes mellitus: The BLOOM-DM study [38]	Obesity	294	2012	42.0	RCT	Type 2	Lorcaserin	IWQOL-LITE
Bariatric surgery versus intensive medical therapy for diabetes – 5-year outcomes [39]	New England Journal of Medicine	293	2017	146.5	RCT	Type 2	Bariatric Surgery, Intensive Medical Therapy	RAND-36
Bariatric-metabolic surgery versus conventional medical treatment in obese patients with type 2 diabetes: 5-year follow-up of an open-label, single-centre, randomized controlled trial [40]	Lancet	289	2015	72.3	RCT	Type 2	Bariatric-metabolic Surgery	RAND-36

RCT: Randomized controlled trials, SF-36 = Short-form 36, WHOQOL-BREF = WHO Quality of Life-BREF, MOS = Medical Outcomes Studies; IWQOL-LITE = Impact of Weight on Quality of Life-LITE.

**Table 3:** Ten research topics classified by Latent Dirichlet Allocation.

Rank	Research Topics	n	Percent
Topic 1	Community-, family-, and telehealth-based interventions to improve self-management and self-efficacy	187	26.7%
Topic 2	Lifestyle (e.g., physical activity and dietary) interventions in people with diabetes	124	17.7%
Topic 3	Interventions address comorbidities in people with diabetes	107	15.3%
Topic 4	Education-based interventions on different aspects of the disease	74	10.6%
Topic 5	Pharmacological treatment to control blood glucose levels	66	9.4%
Topic 6	Pharmacological treatment for diabetic neuropathic pain	52	7.4%
Topic 7	Functional complication interventions	29	4.1%
Topic 8	Interventions addressing mental disorders in people with diabetes	27	3.9%
Topic 9	Surgery and dietary interventions to promote weight loss	18	2.6%
Topic 10	Pharmacological and surgical interventions to address cardiovascular complications	16	2.3%

## References

[1] Cho N.H., Shaw J.E., Karuranga S., Huang Y., da Rocha Fernandes J.D., Ohlrogge A.W., Malanda B. (2018). IDF Diabetes Atlas: Global estimates of diabetes prevalence for 2017 and projections for 2045. Diabetes Res. Clin. Pract..

[2] Smith-Palmer J., Bae J.P., Boye K.S., Norrbacka K., Hunt B., Valentine W.J. (2016). Evaluating health-related quality of life in type 1 diabetes: A systematic literature review of utilities for adults with type 1 diabetes. Clin. Outcomes Res. Ceor.

[3] World Health Organization Diabetes: Fact Sheet. https://www.who.int/news-room/fact-sheets/detail/diabetes.

[4] Baena-Diez J.M., Penafiel J., Subirana I., Ramos R., Elosua R., Marin-Ibanez A., Guembe M.J., Rigo F., Tormo-Diaz M.J., Moreno-Iribas C. (2016). Risk of Cause-Specific Death in Individuals With Diabetes: A Competing Risks Analysis. Diabetes Care.

[5] (2018). Global, regional, and national disability-adjusted life-years (DALYs) for 359 diseases and injuries and healthy life expectancy (HALE) for 195 countries and territories, 1990–2017: A systematic analysis for the Global Burden of Disease Study 2017. Lancet.

[6] Jing X., Chen J., Dong Y., Han D., Zhao H., Wang X., Gao F., Li C., Cui Z., Liu Y. (2018). Related factors of quality of life of type 2 diabetes patients: A systematic review and meta-analysis. Health Qual. Life Outcomes.

[7] Saleh F., Ara F., Mumu S.J., Hafez M.A. (2015). Assessment of health-related quality of life of Bangladeshi patients with type 2 diabetes using the EQ-5D: A cross-sectional study. BMC Res. Notes.

[8] Costanza R., Fisher B., Ali S., Beer C., Bond L., Boumans R., Danigelis N.L., Dickinson J., Elliott C., Farley J. (2007). Quality of life: An approach integrating opportunities, human needs, and subjective well-being. Ecol. Econ..

[9] Galloway S., Bell D., Hamilton C., Scullion A. (2006). Quality of Life and Well-Being: Measuring the Benefits of Culture and Sport: Literature Review and Thinkpiece.

[10] Cummins R.A. (1998). Quality of Life Definition and Terminology: A Discussion Document from the International Society for Quality of Life Studies.

[11] WHOQoL Group (1993). Study protocol for the World Health Organization project to develop a Quality of Life assessment instrument (WHOQOL). Qual. Life Res..

[12] Zhang X., Norris S.L., Chowdhury F.M., Gregg E.W., Zhang P. (2007). The effects of interventions on health-related quality of life among persons with diabetes: A systematic review. Med. Care.

[13] Luscombe F.A. (2000). Health-related quality of life measurement in type 2 diabetes. Value Health J. Int. Soc. Pharm. Outcomes Res..

[14] Watkins K., Connell C.M. (2004). Measurement of health-related QOL in diabetes mellitus. Pharmacoeconomics.

[15] Cai H., Li G., Zhang P., Xu D., Chen L. (2017). Effect of exercise on the quality of life in type 2 diabetes mellitus: A systematic review. Qual. Life Res..

[16] Magwood G.S., Zapka J., Jenkins C. (2008). A Review of Systematic Reviews Evaluating Diabetes Interventions. Diabetes Educ..

[17] Ayadurai S., Hattingh H.L., Tee L.B.G., Md Said S.N. (2016). A Narrative Review of Diabetes Intervention Studies to Explore Diabetes Care Opportunities for Pharmacists. J. Diabetes Res..

[18] Massey C.N., Feig E.H., Duque-Serrano L., Wexler D., Moskowitz J.T., Huffman J.C. (2019). Well-being interventions for individuals with diabetes: A systematic review. Diabetes Res. Clin. Pract..

[19] Emami Z., Hariri N., Khamseh M.E., Nooshinfard F. (2018). Mapping diabetes research in Middle Eastern countries during 2007–2013: A scientometric analysis. Med. J. Islamic Repub. Iran.

[20] Rasolabadi M., Khaledi S., Ardalan M., Kalhor M.M., Penjvini S., Gharib A. (2015). Diabetes Research in Iran: A Scientometric Analysis of Publications Output. Acta Inform. Med..

[21] Bruggmann D., Richter T., Klingelhofer D., Gerber A., Bundschuh M., Jaque J., Groneberg D.A. (2016). Global architecture of gestational diabetes research: Density-equalizing mapping studies and gender analysis. Nutr. J..

[22] Ramin S., Gharebaghi R., Heidary F. (2015). Scientometric Analysis and Mapping of Scientific Articles on Diabetic Retinopathy. Med. Hypothesisdiscov. Innov. Ophthalmol. J..

[23] Sweileh W.M. (2018). Analysis of global research output on diabetes depression and suicide. Ann. Gen. Psychiatry.

[24] Tabatabaei-Malazy O., Ramezani A., Atlasi R., Larijani B., Abdollahi M. (2016). Scientometric study of academic publications on antioxidative herbal medicines in type 2 diabetes mellitus. J. Diabetes Metab. Disord..

[25] Martín-Martín A., Orduna-Malea E., Delgado López-Cózar E. (2018). Coverage of highly-cited documents in Google Scholar, Web of Science, and Scopus: A multidisciplinary comparison. Scientometrics.

[26] Clarivate Analytics. Web of Science databases. https://clarivate.com/products/web-of-science/databases/.

[27] Krzywinski M., Schein J., Birol I., Connors J., Gascoyne R., Horsman D., Jones S.J., Marra M.A. (2009). Circos: An information aesthetic for comparative genomics. Genome Res..

[28] Valle D., Albuquerque P., Zhao Q., Barberan A., Fletcher R.J. (2018). Extending the Latent Dirichlet Allocation model to presence/absence data: A case study on North American breeding birds and biogeographical shifts expected from climate change. Glob. Chang. Biol..

[29] Chen C., Zare A., Trinh H.N., Omotara G.O., Cobb J.T., Lagaunne T.A. (2017). Partial Membership Latent Dirichlet Allocation for Soft Image Segmentation. IEEE Trans. Image Process..

[30] Lu H.M., Wei C.P., Hsiao F.Y. (2016). Modeling healthcare data using multiple-channel latent Dirichlet allocation. J. Biomed. Inf..

[31] Gross A., Murthy D. (2014). Modeling virtual organizations with Latent Dirichlet Allocation: A case for natural language processing. Neural. Netw..

[32] Crowther C.A., Hiller J.E., Moss J.R., McPhee A.J., Jeffries W.S., Robinson J.S. (2005). Effect of Treatment of Gestational Diabetes Mellitus on Pregnancy Outcomes. N. Engl. J. Med..

[33] Backonja M., Beydoun A., Edwards K.R., Schwartz S.L., Fonseca V., Hes M., LaMoreaux L., Garofalo E. (1998). Gabapentin for the symptomatic treatment of painful neuropathy in patients with diabetes mellitus: A randomized controlled trial. JAMA.

[34] Schauer P.R., Bhatt D.L., Kirwan J.P., Wolski K., Brethauer S.A., Navaneethan S.D., Aminian A., Pothier C.E., Kim E.S.H., Nissen S.E. (2014). Bariatric Surgery versus Intensive Medical Therapy for Diabetes—3-Year Outcomes. N. Engl. J. Med..

[35] Weinberger M., Oddone E.Z., Henderson W.G. (1996). Does increased access to primary care reduce hospital readmissions? Veterans Affairs Cooperative Study Group on Primary Care and Hospital Readmission. N. Engl. J. Med..

[36] Davies M.J., Heller S., Skinner T.C., Campbell M.J., Carey M.E., Cradock S., Dallosso H.M., Daly H., Doherty Y., Eaton S. (2008). Effectiveness of the diabetes education and self management for ongoing and newly diagnosed (DESMOND) programme for people with newly diagnosed type 2 diabetes: Cluster randomised controlled trial. BMJ.

[37] Harati Y., Gooch C., Swenson M., Edelman S., Greene D., Raskin P., Donofrio P., Cornblath D., Sachdeo R., Siu C.O. (1998). Double-blind randomized trial of tramadol for the treatment of the pain of diabetic neuropathy. Neurology.

[38] Watson C.P.N., Moulin D., Watt-Watson J., Gordon A., Eisenhoffer J. (2003). Controlled-release oxycodone relieves neuropathic pain: A randomized controlled trial in painful diabetic neuropathy. Pain.

[39] O’Neil P.M., Smith S.R., Weissman N.J., Fidler M.C., Sanchez M., Zhang J., Raether B., Anderson C.M., Shanahan W.R. (2012). Randomized Placebo-Controlled Clinical Trial of Lorcaserin for Weight Loss in Type 2 Diabetes Mellitus: The BLOOM-DM Study. Obesity.

[40] Schauer P.R., Bhatt D.L., Kirwan J.P., Wolski K., Aminian A., Brethauer S.A., Navaneethan S.D., Singh R.P., Pothier C.E., Nissen S.E. (2017). Bariatric Surgery versus Intensive Medical Therapy for Diabetes—5-Year Outcomes. N. Engl. J. Med..

[41] Mingrone G., Panunzi S., De Gaetano A., Guidone C., Iaconelli A., Nanni G., Castagneto M., Bornstein S., Rubino F. (2015). Bariatric-metabolic surgery versus conventional medical treatment in obese patients with type 2 diabetes: 5 year follow-up of an open-label, single-centre, randomised controlled trial. Lancet.

[42] Satterfield D.W., Volansky M., Caspersen C.J., Engelgau M.M., Bowman B.A., Gregg E.W., Geiss L.S., Hosey G.M., May J., Vinicor F. (2003). Community-Based Lifestyle Interventions to Prevent Type 2 Diabetes. Diabetes Care.

[43] Shrestha P., Ghimire L. (2012). A review about the effect of life style modification on diabetes and quality of life. Glob. J. Health Sci..

[44] Trikkalinou A., Papazafiropoulou A.K., Melidonis A. (2017). Type 2 diabetes and quality of life. World J. Diabetes.

[45] Liu L., Jiao J.H., Chen L. (2011). Bibliometric study of diabetic retinopathy during 2000–2010 by ISI. Int. J. Ophthalmol..

[46] Somogyi A., Schubert A.J.S. (2005). Correlation between national bibliometric and health indicators: The case of diabetes. Scientometrics.

[47] Khanal P. (2017). Bringing all together for research capacity building in LMICs. Lancet Glob. Health.

[48] ESSENCE on Health Research (2014). Seven Principle for Strengthening Research Capacity in Low-and-middle-income Countries: Simple Ideas in a Complex World.

[49] Ali N., Hill C., Kennedy A., IJsselmuiden C. (2006). What Factors Influence National Health Research Agendas in Low and Middle Income Countries.

[50] Baghaei Lakeh A., Ghaffarzadegan N. (2017). Global Trends and Regional Variations in Studies of HIV/AIDS. Sci. Rep..

